# Dosimetric effects of air pocket sizes in MammoSite treatment as accelerated partial breast irradiation for early breast cancer

**DOI:** 10.1120/jacmp.v11i1.2932

**Published:** 2009-12-23

**Authors:** Y. Jessica Huang, Melissa Blough

**Affiliations:** ^1^ Department of Radiation Oncology University of Utah Salt Lake City UT; ^2^ Radiation Oncology Department START Center for Cancer Care San Antonio TX; ^3^ Department of Radiology University of Texas Health Science Center San Antonio TX 78229 USA

**Keywords:** Brachytherapy, MammoSite, air cavity, Monte Carlo algorithm

## Abstract

MammoSite brachytherapy system has been used as one of the accelerated partial breast irradiation (APBI) techniques since 2002. The clinical results from several clinical institutions had shown comparable treatment efficacy, cosmesis, and toxicity to other APBI techniques. During MammoSite treatment, air cavities had been one of the primary issues causing treatment cancellation or delay. With the tolerance of the air volume less than 10% of the total Planning Target Volume (PTV) set, there is still no data available to show the actual dose delivered to the breast tissue with the existence of the air pocket. In this paper, Monte Carlo N‐Particle version 5 (MCNP5) was used to model a hypothesis MammoSite phantom with different sizes of air pockets, and compared to the calculation results from the treatment planning system (TPS) without heterogeneous corrections. It was found that without heterogeneous corrections, the difference between the TPS and MCNP5 calculations in the air cavity surface doses and PTV point doses can be up to 2.02% and 3.61%, respectively, using the balloon and air pocket size combinations calculated in this paper. Based on the distance from the point of interest to the balloon surface, an approximate dose can be calculated using the linear relationship found in this study. These equations provide a quick and simple way to predict the actual dose delivered to the breast soft tissue located within the PTV. With the equation applied to the dose from the TPS, the dose error caused by the air pocket during MammoSite treatment can be reduced to a minimum.

PACS number: 87.53.Jw

## I. INTRODUCTION

In the treatment of early‐stage breast cancer, MammoSite (Cytyc Corporation, Marlborough, MA) has been used as one of the accelerated partial breast irradiation (APBI) techniques after breast‐conserving surgery.^(^ ^1^ ^,^ ^2^ ^)^ Several different techniques with similar rationale have been developed in recent years, such as Contura (SenoRx Inc., Los Angeles, CA) and SAVI (Cianna Medical, Aliso Viejo, CA); however, only limited clinical experience is available^(^ ^3^ ^,^ ^4^ ^)^ With three to five years of clinical results reported, it has been shown that MammoSite is an effective treatment modality with comparable reoccurrence rates with conventional whole breast radiation therapy and interstitial brachytherpay^(^ ^5^ ^,^ ^6^ ^,^ ^7^ ^)^ The MammoSite applicator is a single catheter with an inflatable balloon at its distal end that can be placed in the tumor bed cavity that has been resected during surgery.^(^ ^8^ ^,^ ^9^ ^)^ The treatment is performed by delivering the Ir‐192 high‐dose‐rate radioactive source through the center lumen of the catheter by a computer‐controlled remote afterloader while the balloon is inflated in the tumor bed cavity.

In the MammoSite treatment, it has been found that air cavities occasionally exist and can be seen and measured in CT images. Reasons for the air cavity may include: (1) air trapped during the placement of the balloon in the surgical process, (2) non‐spherical resection space, or (3) insufficient balloon filling. Based on clinical experience, about 90% of the patients have air cavities when imaged two days after the balloon placement. The acceptance level for the air cavity volume is less than 10% of the planning target volume (PTV).^(9)^ Although the acceptable amount of air in the MammoSite treatment has been determined, the dose effect due to the air cavity is not taken into account in the treatment planning system (TPS). As modern brachytherapy TPS considers patient anatomy as full‐scattered homogeneous water medium, the TPS incorrectly models irregular patient geometry and any other inhomogeneous factors in the volume of interest.^(10)^ Therefore, heterogeneities like air cavities in MammoSite treatments may cause dose errors to the tissue located near the air‐tissue interface due to lack of attenuation and inverse square corrections. This study is designed to quantify dose errors occurring at the interface of the air cavity and PTV point that has been dislocated by the air cavity in MammoSite treatments with Monte Carlo calculations in order to fully understand the dosimetric effects from different sizes of air cavities on various balloon sizes.

Due to different causes of the air cavities and the geometry of the MammoSite balloon, the air cavities may occur at all directions around the balloon surface in any irregular shapes. To understand the dose effects resulting from various balloon and air cavity size combinations, anisotropic effect was eliminated by placing the air cavity only at the transverse axis of the balloon. Also, only hemisphere shaped air cavities were simulated in the Monte Carlo calculation to simplify the model.

## II. MATERIALS AND METHODS

Monte Carlo N‐Particle version 5 (MCNP5, Los Alamos National Laboratory, Los Alamos, NM) was used in this study. MCNP5 provides users the ability to develop a three‐dimensional geometrical model to simulate all material boundaries, their compositions, and the source locations. With the three‐dimensional model being established, MCNP5 can simulate and track a number of randomly generated particle histories. The simulation process includes incoherent and coherent scattering, K, L‐shell characteristic X‐ray emission after photoelectric absorption, and pair production absorption with local emission of annihilation radiation and bremsstrahlung for photons. In this study, tally F6 was used to calculate dose depositions. The F6 tally scores the energy deposition averaged over a cell with the unit of MeV/g.^(^ ^11^ ^,^ ^12^ ^)^ The cut‐off energy was set at 1 keV. The MC simulations were stopped when the statistical uncertainty reached a value of less than 1%.

The microSelectron I192rHDR source (Nucletron Corp., Veenendaal, The Netherlands) was used for MCNP5 modeling. For MammoSite balloon, two clinical available balloon physical sizes, 4–5 cm and 5–6 cm were simulated. With different balloon filling volume, the width and length of the balloon changes asymmetrically. Since this study was designed to observe the effect of various air cavity sizes on different sizes of the balloon, only the smallest and largest balloon sizes in both 4–5 cm and 5–6 cm were used. The width and length information of each modeled balloon size is listed in Table 1. For each balloon size, four air cavity sizes were modeled in the MCNP5. Water was used in the MCNP5 modeling for balloon filling and to represent breast soft tissue surrounding the balloon. Based on clinical experiences and the criterion of less than 10% of the PTV, the air cavity sizes used in this study ranged from 0.25 cm to 2.0 cm. Except for the normal range of air cavity sizes (0.25 cm to 1.5 cm), a cavity size of close to clinical acceptable value of 10% for each balloon size were also simulated. Table 2 lists all of the balloon and air cavity size combinations calculated in this study and their volume ratio results. To validate the MCNP5 modeling accuracy, TLD measurement in a plastic water phantom (34 cc balloon with 0.5 cm radius air cavity) was performed. For other balloon sizes, the TPS dose results were used to benchmark the MCNP5 modeling.

**Table 1:** MammoSite balloon size information.

	*MammoSite Nominal Fill Volume (cc)*	*Width (cm)*	*Length (cm)*
	34 cc	4.00	4.00
4‐5 cm MammoSite Balloon	70 cc (S)	5.10	4.65
	70 cc (L)	4.87	5.11
5‐6 cm MammoSite Balloon	125 cc	5.90	5.73

To differentiate between two 70 cc balloons in two different balloon sizes, 70 cc (S) and 70 cc (L) are used in this study. 70 cc (S) is for 4–5 cm balloon and 70 cc (L) is for 5–6 cm balloon.

**Table 2:** Air cavity volume ratio.

*Balloon Physical Size*	*Fill Volume*	*Air Cavity Size (radius, in cm)*	*Volume Ratio*
		0.25	0.04%
	34 cc	0.50	0.36%
		1.00	3.12%
		1.44	9.98%
4‐5 cm MammoSite Balloon			
		0.50	0.24%
	70 cc (S)	1.00	2.04%
		1.50	7.32%
		1.66	10.11%
		0.50	0.26%
	70 cc (L)	1.00	2.21%
		1.50	7.96%
		1.62	10.18%
5‐6 cm MammoSite Balloon			
		0.50	0.18%
	125 cc	1.00	1.57%
		1.50	5.59%
		2	9.97%

Sixteen size combinations were used in this study. The volume ratio was calculated by dividing the air cavity volume by the PTV of each balloon size.

**Figure 1:**
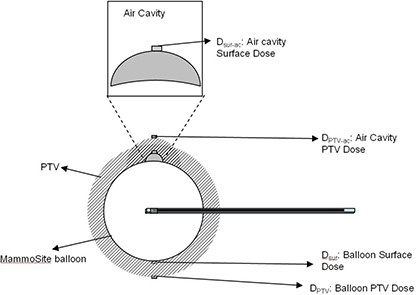
MammoSite balloon and air cavity in the MCNP5 model. Dose points of interest $D_{\text{sur}},\;D_{\text{sur‐ac}},\;D_{\text{PTV}}$, and $D_{\text{PTV‐ac}}$ are also shown in the sketch. $D_{\text{sur}}$ and $D_{\text{sur‐ac}}$ are balloon surface and air cavity surface doses, and $D_{\text{PTV}}$ and $D_{\text{PTV‐ac}}$ are PTV and air cavity PTV doses. The shadowed area is the PTV (i.e. the area within 1 cm from the balloon surface).

Four dose points of interests are shown in Fig. 1. Balloon surface $\left(D_{\text{sur}}\right)$ and PTV doses $\left(D_{\text{ptv}}\right)$ are homogeneous dose points. These two dose points represent delivered doses in optimal treatment setting and their doses should be close to predicted doses in the TPS. Therefore, these two points also served as validation points for using MCNP5 to calculate MammoSite treatment. Air cavity surface $\left(D_{\text{sur‐ac}}\right)$ and air cavity PTV doses $\left(D_{\text{PTV‐ac}}\right)$ are dose points with air cavity interference and their doses were calculated with inhomogeneity correction using MCNP5 simulation. To observe the dose differences occurring at the breast tissue located next to the balloon, a comparison between balloon surface $\left(D_{\text{sur}}\right)$ and air cavity surface $\left(D_{\text{sur‐ac}}\right)$ doses was performed. Also, the PTV doses were compared between balloon PTV $\left(D_{\text{ptv}}\right)$ and air cavity PTV $\left(D_{\text{PTV‐ac}}\right)$ results.

**Figure 2:**
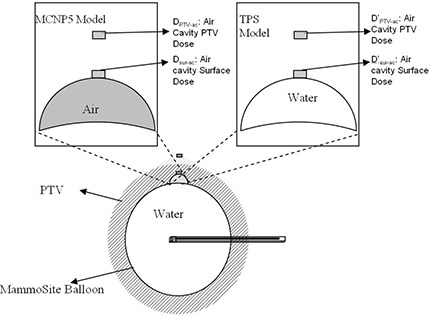
Two models used in the MCNP5 calculations. The MCNP5 model has air filled in the air cavity space; the TPS model has water filled in the space. $D_{\text{sur‐ac}}$ and $D_{\text{sur‐ac}}^{'}$ and are used to show air cavity surface doses, and $D_{\text{PTV‐ac}}$ and $D_{\text{sur}-\text{ac}}^{,}$ are used for air cavity PTV doses.

Since the TPS calculated doses to the air cavity surface and air cavity PTV point without considering the inhomogeneity effect from the air, the TPS dose results $D_{\text{sur}-\text{ac}}^{,}$ and $D_{\text{PTV}-\text{ac}}^{,}$, can be represented with MCNP5 modeling the air cavity filled with water. Therefore, two models were used in the MCNP5 calculations, one was MCNP5 model with the air cavity filled with air, and another one was TPS model with the air cavity filled with water. As shown in Fig. 2, $D_{\text{sur‐ac}}$ and $D_{\text{sur‐ac}}$ and $D_{\text{PTV‐ac}}$ and $D_{\text{sur}-\text{ac}}^{,}$ are used to compare the differences between results from MCNP5 and TPS calculations in inhomogeneous environment. These comparisons show the possible error caused by TPS without inhomogeneous corrections.

## III. RESULTS

**Figure 3:**
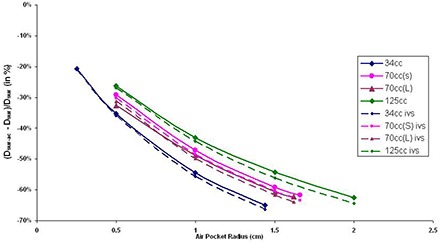
Balloon surface $\left(D_{\text{sur}}\right)$ and air cavity surface $\left(D_{\text{sur‐ac}}\right)$ dose differences (solid lines) and inverse square factor (dashed lines) results with various air cavity sizes (in radius) in different balloon sizes. The y‐axis is calculated by $\left(D_{\text{sur‐ac}}-D_{\text{sur}})/D_{\text{sur}}\;(\%\right)$.

TLD measurements and TPS results comparisons both showed MCNP5 can be used to model the calculations of air cavity in MammoSite treatment. The detailed results are not listed in this paper. The MCNP5 results for 34 cc, 70 cc (S), 70 cc (L), and 125 cc balloon with various air cavity sizes from radius 0.25 to 2.0 cm are listed in Table 3 based on the point locations. Significant lower doses are found in air cavity surface and air cavity PTV point doses. The resulting difference between balloon surface $\left(D_{\text{sur}}\right)$ and air cavity surface $\left(D_{\text{sur‐ac}}\right)$ represent the dose differences at the breast tissue junction caused by the air cavity. With the air cavity pushing out the breast tissue, $D_{\text{sur‐ac}}$ doses are lower than $D_{\text{sur}}$ doses due to the longer distance between the tissue and the source. The calculated percentage differences between $D_{\text{sur}}$ and $D_{\text{sur‐ac}}$ are shown in solid lines in Fig. 3, according to the air cavity size (radius in cm) and balloon size (cc). Depending on the balloon and air cavity size, the percentage differences between $D_{\text{sur}}$ and $D_{\text{sur‐ac}}$ ranged from −20.65% to −64.97%. The negative signs represented lower doses received at the air cavity surface, $D_{\text{sur‐ac}}$. It is found that with larger air cavity size, the dose differences between expected surface dose and air cavity surface dose increase. Also, depending on the balloon size, the longer the distance between the source and the dose point, the more significant the dose differences become.

**Table 3:** Dose point results in different balloon and air cavity size combinations. The unit is in cGy.

*34 cc*	*Air Cavity Radius*			
	*0.25 cm*	*0.50 cm*	*1.00 cm*	*1.44 cm*
Balloon Surface $\left(D_{\text{sur}}\right)$	760.7	761.4	766.6	769.5
Air Cavity Surface ${(D}_{\text{sur}-\text{ac}})$	603.3	492.6	346.5	266.1
PTV Point $\left(D_{\text{PTV}}\right)$	336.2	337.4	337.4	337.9
Air Cavity PTV Point ${(D}_{\text{PVT}-\text{ac}})$	286.8	251.7	196.6	163.2
*70 cc (S)*	*Air Cavity Radius*			
	*0.50 cm*	*1.00 cm*	*1.50 cm*	*1.66 cm*
Balloon Surface $\left(D_{\text{sur}}\right)$	670.0	670.1	671.3	669.3
Air Cavity Surface ${(D}_{\text{sur}-\text{ac}})$	475.3	354.6	274.7	256.7
PTV Point $\left(D_{\text{PTV}}\right)$	340.3	340.8	341.5	340.2
Air Cavity PTV Point ${(D}_{\text{PVT}-\text{ac}})$	267.2	217.3	179.9	169.0
*70 cc (L)*	*Air Cavity Radius*			
	*0.50 cm*	*1.00 cm*	*1.50 cm*	*1.62 cm*
Balloon Surface $\left(D_{\text{sur}}\right)$	679.4	681.7	681.5	675.8
Air Cavity Surface ${(D}_{\text{sur}-\text{ac}})$	470.2	347.4	267.1	255.0
PTV Point $\left(D_{\text{PTV}}\right)$	345.4	341.1	337.2	334.9
Air Cavity PTV Point ${(D}_{\text{PVT}-\text{ac}})$	262.5	209.3	173.1	165.6
*125 cc*	*Air Cavity Radius*			
	*0.50 cm*	*1.00 cm*	*1.50 cm*	*2.0 cm*
Balloon Surface $\left(D_{\text{sur}}\right)$	614.8	616.3	615.5	641.5
Air Cavity Surface ${(D}_{\text{sur}-\text{ac}})$	454.0	351.4	281.0	230.5
PTV Point $\left(D_{\text{PTV}}\right)$	339.4	340.2	340.4	338.1
Air Cavity PTV Point ${(D}_{\text{PVT}-\text{ac}})$	271.9	225.0	189.6	161.2

**Figure 4:**
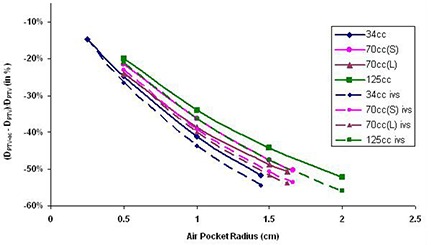
PTV dose $\left(D_{\text{PTV}}\right)$ and air cavity PTV $\left(D_{\text{PTV‐ac}}\right)$ dose differences and inverse square factor results with various air cavity sizes (in radius) in different balloon sizes. The y‐axis is calculated by $\left(D_{\text{PTV‐ac}}-D_{\text{PTV}})/D_{\text{PTV}}\;(\%\right)$.

PTV dose points were located 1 cm from the balloon surface $\left(D_{\text{PTV}}\right)$ and the air cavity surface $\left(D_{\text{PTV‐ac}}\right)$. Because of the dislocation of surrounding tissue by the air cavity, the air cavity PTV doses $\left(D_{\text{PTV‐ac}}\right)$ are lower than PTV doses $\left(D_{\text{PTV}}\right)$. The resulting percentage differences between $D_{\text{PTV}}$ and $D_{\text{PTV‐ac}}$ are plotted against the air cavity radius (Fig. 4) based on the balloon fill volume. The two PTV dose points differences range between −14.71% and −52.32%, where the negative signs are used to show that $D_{\text{PTV‐ac}}$ received less dose than $D_{\text{PTV}}$. Similar to the results found with the surface dose comparison ($D_{\text{sur}}$ and $D_{\text{sur‐ac}}$), it can also be shown that the larger air cavity size and longer source to dose point distance will cause increasing differences between PTV dose points ($D_{\text{PTV}}$ and $D_{\text{PTV‐ac}}$).

**Figure 5:**
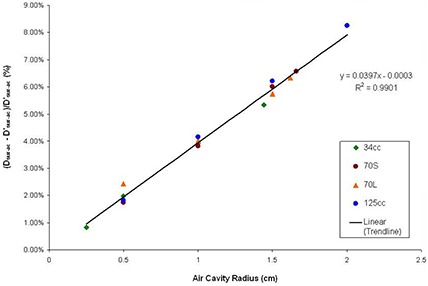
Air cavity surface dose comparisons between MCNP5 and TPS model. The x‐axis is the air cavity radius in cm, and the y‐axis is the difference between the two models, calculated by ($D_{\text{sur}-\text{ac}}^{,}$ – $D_{\text{sur}-\text{ac}}^{,}$)/ $D_{\text{sur}-\text{ac}}^{,}$ (%). The data points are results from this study and the line shows the linear regression line. The linear regression line equation and $R^{2}$ are also shown.

The dose results from TPS models in the MCNP5 calculations are listed in Table 4 as point $D_{\text{sur}-\text{ac}}^{,}$ and $D_{\text{sur}-\text{ac}}^{,}$. As described before, these two models were used to compare the dose differences caused by calculations performed with and without inhomogeneous corrections. The TPS model calculated doses at air cavity surface and 1 cm from air cavity surface using the TPS hypothesis, which excluded inhomogeneity in the air cavity calculation. These results show higher doses received at the air cavity surface and air cavity PTV point when inhomogeneity corrections were taken into account. The percentage differences between air cavity surface doses in different models, MCNP5 model and TPS model, are plotted against the radius of the air cavity based on the balloon filling sizes (Fig. 5). For all of the situations studied, air cavity surface doses in MCNP5 model $\left(D_{\text{sur‐ac}}\right)$ results are higher than TPS model ($D_{\text{sur}-\text{ac}}^{,}$) results ranging from 0.82% to 8.25%.

**Table 4:** Two simulation models comparison: MCNP5 model with air cavity filled with air, and TPS model with air cavity filled with water.

*34 cc*	*Air Cavity Radius*				
		*0.25 cm*	*0.50 cm*	*1.00 cm*	*1.44 cm*
	MCNP5 ${(D}_{\text{sur}-\text{ac}})$	603.3 cGy	492.6 cGy	346.5 cGy	266.1 cGy
Air Cavity Surface	TPS ($D_{\text{sur}-\text{ac}}^{,}$)	598.3 cGy	483.0 cGy	333.4 cGy	252.6 cGy
	MCNP5 ${(D}_{\text{PVT}-\text{ac}})$	286.8 cGy	251.7 cGy	196.6 cGy	161.3 cGy
Air Cavity PTV Point	TPS ($D_{\text{sur}-\text{ac}}^{,}$)	281.7cGy	243.8 cGy	184.7 cGy	149.9 cGy
*70 cc (S)*	*Air Cavity Radius*				
		*0.50 cm*	*1.00 cm*	*1.50 cm*	*1.66 cm*
	MCNP5 ${(D}_{\text{sur}-\text{ac}})$	475.3 cGy	354.6 cGy	274.7 cGy	256.7 cGy
Air Cavity Surface	TPS ($D_{\text{sur}-\text{ac}}^{,}$)	467.2 cGy	341.7 cGy	259.1 cGy	241.1 cGy
	MCNP5 ${(D}_{\text{PVT}-\text{ac}})$	267.2 cGy	217.3 cGy	179.9 cGy	169.0 cGy
Air Cavity PTV Point	TPS ($D_{\text{sur}-\text{ac}}^{,}$)	260.1 cGy	205.4 cGy	165.9 cGy	155.0 cGy
*70 cc (L)*	*Air Cavity Radius*				
		*0.50 cm*	*1.00 cm*	*1.50 cm*	*1.62 cm*
	MCNP5 ${(D}_{\text{sur}-\text{ac}})$	470.2 cGy	347.5 cGy	267.2 cGy	255.0 cGy
Air Cavity Surface	TPS ($D_{\text{sur}-\text{ac}}^{,}$)	458.7 cGy	333.9 cGy	252.6 cGy	239.7 cGy
Air Cavity PTV Point	MCNP5 ${(D}_{\text{PVT}-\text{ac}})$	262.5 cGy	209.1 cGy	173.1 cGy	165.6 cGy
	TPS ($D_{\text{sur}-\text{ac}}^{,}$)	254.3 cGy	197.5 cGy	160.1 cGy	152.0 cGy
*125 cc*	*Air Cavity Radius*				
		*0.50 cm*	*1.00 cm*	*1.50 cm*	*2.00 cm*
	MCNP5 ${(D}_{\text{sur}-\text{ac}})$	453.9 cGy	351.6 cGy	280.8 cGy	230.5 cGy
Air Cavity Surface	TPS ($D_{\text{sur}-\text{ac}}^{,}$)	445.7 cGy	337.3 cGy	264.5 cGy	212.8 cGy
	MCNP5 ${(D}_{\text{PVT}-\text{ac}})$	272.0 cGy	225.1 cGy	189.7 cGy	161.2 cGy
Air Cavity PTV Point	TPS ($D_{\text{sur}-\text{ac}}^{,}$)	263.8 cGy	212.5 cGy	174.8 cGy	145.5 cGy

**Figure 6:**
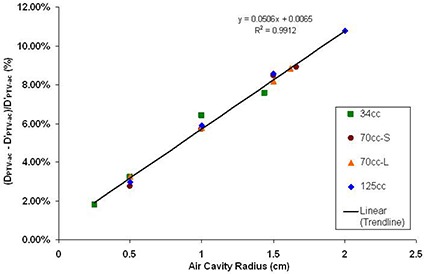
Air cavity PTV dose comparisons between MCNP5 and TPS model. The x‐axis is the air cavity radius in cm, and the y‐axis is the difference between the two models, calculated by ($D_{\text{PTV}-\text{ac}}^{,}$ – $D_{\text{sur}-\text{ac}}^{,}$)/ $D_{\text{sur}-\text{ac}}^{,}$ (%). The resulting linear regression line, its equation and $R^{2}$ are shown with the data points on the graph.

The differences between air cavity PTV doses in the two different calculation models are summarized in Fig. 6. Similar to air cavity surface dose results (Fig. 5), air cavity PTV doses in MCNP5 model $\left(D_{\text{PTV‐ac}}\right)$ are higher than doses in TPS model ($D_{\text{sur}-\text{ac}}^{,}$) under studied situations, ranging between 1.81% and 10.75%. From Figs. 5 and 6, it is found that for both air cavity surface dose and air cavity PTV dose, increasing the air cavity sizes will increase the differences between doses calculated in MCNP5 and TPS models. Changing balloon size, however, doesn't have significant effect using the two calculation models.

## IV. DISCUSSION

When comparing air cavity surface doses $\left(D_{\text{sur‐ac}}\right)$ and balloon surface doses $\left(D_{\text{sur}}\right)$, the geometric distance differences between the two points causes major dose discrepancies (Fig. 3) due to inverse square effect. The same effects are also shown in PTV dose comparisons ($D_{\text{PTV}}$ and $D_{\text{PTV‐ac}}$, Fig. 4). It has been found in this study that an inverse square factor should be applied to account for the dislocation of the breast tissue by the air cavity. The inverse square factor (IVS) can be calculated with the following equation:

(1) $$\text{Inverse}\;\text{square}\;\text{factor}\;(\%)=\;\;\;\;\;\;\;-\left[1-{\left(\frac{r_{b}}{r_{b}+r_{a}}\right)}^{2}\right]*100\%$$

where $r_{a}$ is the radius of the air cavity (cm), and $r_{b}$ is the radius of the balloon (cm).

In Figs. 3 and 4, the dotted line shows the inverse square factor with various air cavity sizes appearing in different balloon sizes. It is found that the differences between $D_{\text{sur}}$ and $D_{\text{sur‐ac}}$ and $D_{\text{PTV}}$ and $D_{\text{PTV‐ac}}$ are all less than the results from inverse square factors. This is because the dose differences occurring between those dose points are the results from both inverse square factors and inhomogeneities from the air cavities. Currently, the majority of the TPS available in the market calculate doses as if the sources are surrounded by water (homogeneous calculations). However, in the presence of low density, and low Z air cavities, the doses are slightly increased by scattering effects from surrounding tissues. With inverse square factor to decrease doses and heterogeneity effects to increase doses, the resulting MCNP5 plots showed less dose discrepancies between dose points ($D_{\text{sur}}-D_{\text{sur‐ac}}$ and $D_{\text{PTV}}-D_{\text{PTV‐ac}})$ than inverse square factor plots. The differences between the inverse square plots and MCNP5 calculations were less than 2.02% in $D_{\text{sur}}-D_{\text{sur‐ac}}$, and less than 3.61% in $D_{\text{PTV}}-D_{\text{PTV‐ac}}$. These values showed that the heterogeneity effects will introduce small dose discrepancies. Therefore, when inhomogeneous correction is not available and only the balloon surface/PTV doses are calculated in the TPS, an inverse square factor should be applied in order to better estimate the air cavity surface $\left(D_{\text{sur‐ac}}\right)$ and air cavity PTV $\left(D_{\text{PTV‐ac}}\right)$ doses when an air cavity appears in a MammoSite treatment delivery.

A second method to estimate the actual dose delivered to the breast tissue when air cavities appear during the MammoSite treatment was found in this study. Since the location of the air cavity can be specified in the TPS, the air cavity surface and PTV doses can be calculated. As has been shown in Figs. 5 and 6, the degree of dose errors approximated a linear function of the air cavity radius. With this linear relationship, it is possible to estimate the dose errors at the air cavity surface and PTV point by applying the linear function according to the balloon sizes. Therefore, if the dose points at the air cavity surface and the air cavity PTV are being calculated in the TPS, it is recommended to use the following two linear approximation equations (LAEQ) to predict the actual delivered point doses:

(2) $$\begin{array}{l} \text{Actual}\;\text{Dose}\;\text{to}\;\text{the}\\ \text{Air}\;\text{Cavity}\;\text{Surface}\;(\text{cGy})=\;\;\;\;\left[(0.0397\times r_{a}-0.0003)\times D_{\text{sur},\text{TPS}}\right]+D_{\text{sur},\text{TPS}} \end{array}$$

(3) $$\begin{array}{l} \text{Actual}\;\text{Dose}\;\text{to}\;\text{the}\\ \text{Air}\;\text{Cavity}\;\text{PTV}\;(\text{cGy})=\;\;\;\;\left[(0.0506\times r_{a}-0.0065)\times D_{\text{PTV},\text{TPS}}\right]+D_{\text{PTV},\text{TPS}} \end{array}$$

where $r_{a}$ is the air cavity radius (cm), $D_{\text{surTPS}}$ is the air cavity surface dose (cGy) calculated by the TPS, and $D_{\text{PTV},\text{TPS}}$ is the air cavity PTV dose (cGy) calculated by the TPS.

After applying LAEQ method described above, it is found that the dose differences between LAEQ results and MCNP5 model results can be achieved to be within 0.5%. Therefore, comparing with the IVS method, LAEQ will result in better estimated value to air cavity surface and PTV doses. It should also be noted that the usage of either IVS or LAEQ method only estimate situations where the air cavity is close to hemisphere shape and located at the transverse axis. With air cavity located other than the transverse axis, an anisotropic factor should be taken into account in these two equations.

For all of the balloon sizes, this study also simulated air cavity sizes close to 10% of the balloon fill volume (clinical limits for treatment delivery) to show the maximal dose differences caused by air cavity during clinical treatment. When comparing TPS and MCNP5 models, the dose differences can be up to 8.25% for $D_{\text{sur‐ac}}$ and $D_{\text{sur}-\text{ac}}^{,}$; and 10.75% for $D_{\text{PTV‐ac}}$ and $D_{\text{sur}-\text{ac}}^{,}$. These two values show the actual doses delivered to the air cavity and PTV points are higher than those calculated from the TPS. However, with big air cavity sizes, the delivered doses to the points of interests become much lower than prescribed dose, 340 cGy. For example, in the case of 125 cc balloon with 2 cm radius air cavity (9.97%), the dose to the air cavity PTV $\left(D_{\text{PTV‐ac}}\right)$ becomes 230.5 cGy (47.4%). Therefore, even though the dose discrepancies are not significant, clinicians should always carefully evaluate the treatment appropriateness with air cavity due to the large IVS effects, especially when the original cancerous sites can be located close to the dislocated breast tissue.

## V. CONCLUSIONS

This study demonstrates that the currently used air cavity volume of less than 10% criterion is appropriate for MammoSite treatment. When an air cavity presents, it is possible to estimate actual air cavity surface and air cavity PTV doses with heterogeneity correction using Monte Carlo calculations. If inhomogeneity corrections are not available in the TPS, it is recommended to use IVS (Eq. (1)) or LAEQ (Eqs. (2) & (3)) to better estimate the actual delivered doses under limited situations. Extended situations with different air cavity shapes and locations can be studied with Monte Carlo method.
